# Epidemiology of Coronavirus COVID-19: Forecasting the Future Incidence in Different Countries

**DOI:** 10.3390/healthcare8020099

**Published:** 2020-04-15

**Authors:** Johannes Stübinger, Lucas Schneider

**Affiliations:** Department of Statistics and Econometrics, University of Erlangen-Nürnberg, Lange Gasse 20, 90403 Nürnberg, Germany

**Keywords:** Coronavirus, COVID-19, epidemiology, incidence, dynamic time warping, lead-lag effects, forecasting, control strategies, risk management

## Abstract

This paper forecasts the future spread of COVID-19 by exploiting the identified lead-lag effects between different countries. Specifically, we first determine the past relation among nations with the aid of dynamic time warping. This procedure allows an elastic adjustment of the time axis to find similar but phase-shifted sequences. Afterwards, the established framework utilizes information about the leading country to predict the Coronavirus spread of the following nation. The presented methodology is applied to confirmed Coronavirus cases from 1 January 2020 to 28 March 2020. Our results show that China leads all other countries in the range of 29 days for South Korea and 44 days for the United States. Finally, we predict a future collapse of the healthcare systems of the United Kingdom and Switzerland in case of our explosion scenario.

## 1. Introduction

The outbreak of the COVID-19 undoubtedly poses the biggest public health challenge since the Spanish flu in 1918 and 1919 [1]. Multiples states, including Spain, the United States of America, and Portugal, declared the state of emergency following a rapid surge in SARS-COV-2 infections [2,3,4]. According to the Johns Hopkins University [5], the virus already infected 660,706 people and killed 30,862 people worldwide (date: 28 March 2020), overwhelmed hospitals in Italy and brought the global economy to a halt [6,7]. Also in the financial world, COVID-19 caused havoc, resulting in the worst trading day of the S&P 500 (−9.5%) and FTSE 100 (−10.9%) since 1987 [8]. States around the world are taking drastic countermeasures, such as complete lockdowns and social distancing, to contain the spread [9,10].

First cases of COVID-19, which is caused by the Severe Acute Respiratory Syndrome Coronavirus 2 (SARS-CoV-2), were reported on 8 December 2019 in Wuhan, China. Most of the initial patients had exposure to the local Huanan South China seafood market that sells a variety of wild animals, suggesting that the zoonotic Coronavirus crossed the barrier from animal to human at this wet market [11,12,13]. Notable, researcher already described in 2007 the time bomb of combining SARS-CoV-like viruses together with the southern Chinese culture [14]. Over the last two decades, two main epidemics were caused by other two Coronaviruses, namely the Severe Acute Respiratory Syndrome (SARS-CoV) and Middle East Respiratory Syndrome (MERS-CoV) [15,16]. The ticking bomb eventually exploded on 11 March 2020, when the World Health Organization declared COVID-19 as pandemic, the first of its kind caused by a Coronavirus [17].

The dramatic effects of COVID-19 on our daily life and the economy has led to a major scientific interest in this novel virus. At this point in time, multiple substantial questions about this pandemic are still unanswered [18]. Besides medicine, microbiology, and bioinformatics, the outbreak of the COVID-19 also draws attention in the field of epidemiology and statistics. Particular focus within those disciplines lies on time series analysis and forecasting models [19,20,21,22]. With the help of a precise prediction of the further course of development, important countermeasures can be taken in the area of risk management and communication. Surprisingly, the existing literature about forecasting the Coronavirus solely considers individual, country-specific time series in their forecasting models and neglects the lead-lag effects between countries.

This manuscript contributes to the academic world in three ways. First, we develop a novel statistical approach to forecast future developments by taking into account lead-lag effects between different time series. Specifically, the concept of dynamic time warping is employed to determine non-linear relations between nations. Therefore, we are in a position to identify similar, but time-shifted, time sequences. Next, the implemented algorithm predicts the future development of the following time series by exploiting the information about the leading time series. Second, we apply the algorithm to COVID-19 cases of the 10 most affected countries from 1 January 2020 to 28 March 2020. We observe that the underlying methodology is able to detect causal relationships, e.g., China is the worldwide source of the Coronavirus as well as Italy is the forerunner in Europa. Third, we forecast the Coronavirus spread of each country based on the past development of China. Naturally, people and public authorities want to be prepared for all possible scenarios to ensure the best disease prevention and risk management. Therefore, we introduce three possible scenarios, namely, recovery, growth, and explosion of the Coronavirus. We find that an explosion would lead to a collapse of the healthcare systems in the United Kingdom and Switzerland.

The remainder of this paper is structured in the following way. Section 2 introduces our underlying dynamic time warping framework. The optimal causal path algorithm of our analysis approach is described in Section 3. In Section 4, we provide an overview of the underlying data. Section 5 applies the developed algorithm to real-world COVID-19 data. Finally, we summarize our work and give an outlook on future research areas in Section 6.

## 2. Dynamic Time Warping

Measuring similarities of time series possesses a long tradition in both literature and in practice. The vast bulk of existing literature uses classic similarity key figures to quantify the strength of relation [23,24,25,26,27,28]. In concrete terms, these studies measures the similarity between two time series $x=\left(x\left(1\right),\ldots ,x\left(N\right)\right)\in \;R^{N}$ and $y=\left(y\left(1\right),\ldots ,y\left(N\right)\right)\in R^{N}$ by the distance

(1) $$\begin{array}{c} d\left(x,y\right)=\sum_{i=1}^{N}d\left(x\left(i\right),y\left(i\right)\right), \end{array}$$

where d(x(i),y(i)) defines the distance at fixed time *i* (i∈{1,…,N}). An important disadvantage of these measures is that the two time series must have the same length (N=M). Furthermore, the measure shown in Equation (1) is very sensitive to time shifts and misalignments [29]. The concept of dynamic time warping solves these problems by introducing a highly flexible model to identify the relation structure of two given time series $x=\left(x\left(1\right),\ldots ,x\left(N\right)\right)\in R^{N}$ and $y=\left(y\left(1\right),\ldots ,y\left(M\right)\right)\in R^{M}$. In general, it allows an elastic adjustment of the time axis to identify similar but phase-shifted sequences. From the statistical point of view, we specify the similarity between *x* and *y* by

(2) $$\begin{array}{c} c\left(x,y\right)=\sum_{i=1}^{I}c\left(x\left(n_{i}\right),y\left(m_{i}\right)\right), \end{array}$$

where *c* defines the local cost measure and I∈{max(N,M),…,N+M−1}. Dynamic time warping efficiently finds the most appropriate nonlinear mapping by minimizing the measure shown in Equation (2). This method is able to handle time series of different length as well as being robust against migration, noise, and amplitude changes [30]. The concept of dynamic time warping is mainly founded on causal paths. Following [31], a sequence of points $p=(p_{1},\ldots ,p_{I})$ with $p_{i}=\left(n_{i},m_{i}\right)\in \left\{1,\ldots ,N\right\}\times \left\{1,\ldots ,M\right\}$ for i∈{1,…,I} (I∈{max(N,M),…,N+M−1}) is called causal path (warping path) if it meets the following three characteristics:

1. $p_{1}=\left(1,1\right)$ and $p_{I}=\left(N,M\right)$ (Boundary condition).
2. $n_{1}\leq n_{2}\leq \cdots \leq n_{I}$ and $m_{1}\leq m_{2}\leq \cdots \leq m_{I}$ (Monotonicity condition).
3. $p_{i+1}-p_{i}\in \left\{\left(1,0\right),\left(0,1\right),\left(1,1\right)\right\},\;\forall i\in \left\{1,\ldots ,I-1\right\}$ (Step size condition).

Of course, the step size condition implies the monotonicity condition, but this is stated for clarity. We define *P* as the set of all possible causal paths between the given time series *x* and *y*. The total cost of a causal path *p* (p∈P) is determined by

(3) $$\begin{array}{c} c_{p}\left(x,y\right)=\sum_{i=1}^{I}c\left(x\left(n_{i}\right),y\left(m_{i}\right)\right), \end{array}$$

where *c* describes the local cost measure and $c(x\left(n_{i}\right),y\left(m_{i}\right))$ defines the gap between the realizations of *x* at time $n_{i}$ and *y* at time $m_{i}$ (i∈{1,…,I}). Usually, the cost measure is based on the Manhattan distance [32,33,34] or the Euclidean distance [35,36,37]. The optimal causal path $p^{*}$ between the time series *x* and *y* possesses lowest total cost of any possible causal path:

(4) $$\begin{array}{c} p^{*}=\underset{p\in P}{argmin}\;c_{p}\left(x,y\right). \end{array}$$

**Figure 1:**
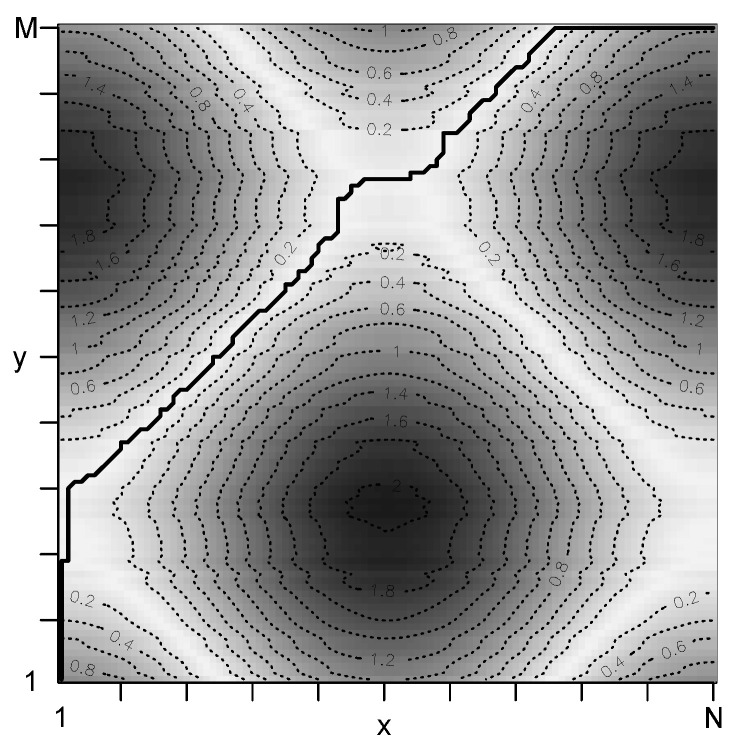
Local costs of two time series *x* and *y* and the optimal warping path $p^{*}$ (solid line). Regions of low cost (high cost) are presented by light colors (dark colors).

The total cost of $p^{*}$ is defined as $c_{p^{*}}\left(x,y\right)$, i.e., the sum of all local costs of $p^{*}$. Figure 1 illustrates the local costs and the identified optimal warping path $p^{*}$ given two time series. Graphically, the sequence of points $p^{*}$ runs along a “valley” of low cost (light colors) and avoids “mountains” of high cost (dark color). In this example, $p^{*}$ is above the diagonal, i.e., the time series *x* leads *y*.

**Figure 2:**
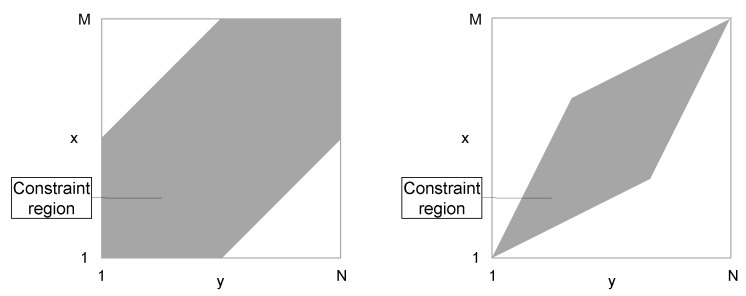
Sakoe–Chiba band (left side) and Itakura parallelogram (right side) with corresponding constraint regions.

In addition to the three path conditions described above, academic studies establish local and global restrictions with the primary purpose of speeding up computing time. Local restrictions vary the step size condition by changing the set of potential steps or preferring certain step directions [38,39,40,41]. Global restrictions aim at limiting the deviation of a causal path from the diagonal–key representatives are the Sakoe-Chiba-Band [42] and the Itakura parallelogram [43] (see Figure 2). However, we avoid local and global restrictions, as both require additional parameter settings and deliever inadequate results in most scenarios [44].

In the 21st century, theoretical research has focused either on the development of a generalized model framework or on the optimization of computing time. In the scope of generalization, [45,46] include the Boltzmann factor proportional to the exponent of the global imbalance of this path. [47] implement a symmetric variant for identifying the time-dependent mapping. Finally, [34] quantifies the optimal lead-lag structure between two time series under the assumption that there is no structural break in the data set. In the scope of optimization, [48] introduces an amendment of the dynamic time warping that employs a higher order representation of the data. Furthermore, [44,49] recursively project an alignment path calculated at a coarse resolution level to the next higher level and then refine it. [50] dynamically exploit the possible existence of an inherent similarity between two time series. Last but not least, [51] launch a memory constrained alignment procedure and [52] use an upper bound estimate to limit less promising warp alignments.

Its outstanding flexibility and adaptability enables research studies to use the dynamic time warping in a wide spectrum of different applications. First, it is employed in speech recognition to compensate non-linear time shifts between two speech patterns as a consequence of different pronunciation [53,54,55]. Most recently, dynamic time warping is mainly used in the field of chemistry [56,57], gesture recognition [58,59], finance [34,60,61], and medicine [62,63].

## 3. Methodology

This section determines the lead-lag relation of two given time series $x\in R^{N}$ and $y\in R^{M}$ and provides a forecasting based on the gained knowledge. Specifically, we i) identify the optimal warping path, ii) determine the lead-lag relation, and iii) predict the future development of the following time series. Following the majority of literature, we define the local cost measure *c* as the absolute difference between $x(n_{i})$ and $y(m_{i})$ (i∈{1,…,I}), see Equation (2).

### 3.1. Step 1: Identify the Optimal Warping Path

First of all, we have to identify the historical non-linear relation between *x* and *y*. Therefore, the local cost matrix is determined, i.e., we calculate all pairwise costs $c(x_{i},y_{j})\;$ ∀i∈{1,…,N},j∈{1,…,M}. Using this matrix, we search for the optimal warping path $p^{*}$, which represents the best possible alignment for the two time series. As mentioned in Section 2, $p^{*}$ has to fulfill the boundary condition, monotonicity condition, and step size condition. Our algorithm is recursive: In each step, we take into account the cost between the affected points and add it to the minimum cost we have found so far. This gives us the optimal distance of two sequences to this position. From a technical point of view, the following recursion scheme is applied:

$$c_{p}\left(x_{i},y_{j}\right):=c\left(x_{i},y_{j}\right)+min\left(c_{p}\left(x_{i-1},y_{j}\right),c_{p}\left(x_{i},y_{j-1}\right),c_{p}\left(x_{i-1},y_{j-1}\right)\right)\;\left(i\in \left\{2,\ldots ,N\right\},j\in \left\{2,\ldots ,M\right\}\right).$$

In the marginal areas, i.e., *i* or *j* equals 1, we adapt this equation by neglecting not available total costs. By the recursive procedure we obtain the optimal warping path $p^{*}$ between *x* and *y*.

### 3.2. Step 2: Determine the Lead-Lag Relation

After finding the optimal causal path, we determine by how many lags *l* time series *x* leads time series *y*—without loss of generality *y* can also lead *x*. For this purpose, the optimal lag *l* is identified by determining the average between the differences of the indices of $p^{*}$. Concretely, we calculate the median, i.e., the value separating the higher half from the lower half of a data sample. Following [34], this procedure supports to receive a robust estimation of the optimal lag because temporally noise terms have almost no influence. In a similar spirit, we calculate the standard deviation of the differences of the indices of $p^{*}$. Consequently, we receive a kind of confidence interval that provides information that the true lag is in the proposed range. Negative values indicate that time series *x* leads time series *y* and vice versa–zero means that they do not influence each other.

### 3.3. Step 3: Forecast the Future Development

Last but not least, we use the information from step 2 to predict the future of the following time series. To be more specific, we know that *x* leads *y* by *l* lags or vice versa. Consequently, the development of the next *l* lags of *y* equals the behaviour of *y* of the last *l* days. Both time series possess a different level which is why we forecast based on cumulative returns of *x* and *y*. Following [64,65], predictions are naturally always associated with uncertainty. Therefore, we distinguish the following three scenarios:

- Recovery: The forecasts are based on the assumption that the development of the following time series will be degressive in the future. The time series *y* increases to a lesser extent in relation to the change in time.
- Growth: This scenario predicts data taking into account that there is a normal development. The currently existing circumstances and conditions are projected into the future.
- Explosion: Forecasts are conducted by assuming that things are getting out of hand. Therefore, the instantaneous rate of change is proportional to the quantity itself.

## 4. Data

**Figure 3:**
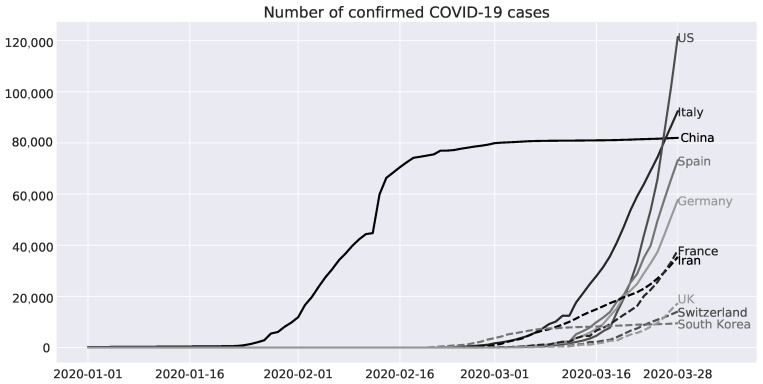
Number of confirmed COVID-19 cases for the 10 most affected countries United States (US), Italy, China, Spain, Germany, France, Iran, United Kingdom (UK), Switzerland, and South Korea from 1 January 2020 to 28 March 2020.

This section provides an overview of past development and the status quo of the COVID-19 spread across different countries (We thank [66] for providing the data). Figure 3 illustrates the confirmed COVID-19 case time series of the ten countries where the disease is most prominent, namely United States, Italy, China, Spain, Germany, France, Iran, United Kingdom, Switzerland, and South Korea. This data set serves as a crucial test for any statistical methodology since it covers 80 % of all COVID-19 cases. It is clearly perceivable that China leads the overall trend. This fact is not surprising as COVID-19 originated in China. Despite the ineffective risk communications of the official authorities, China managed to drastically slow the spread of COVID-19 in March 2020 [67]. Ultimately, China’s confirmed cases got surpassed by Italy and the United States in the same month. This is particularly remarkable under consideration of the population of those countries. China has around 4.26 (23.05) times the population of the United States (Italy) [68]. This could be partially driven by a lack of testing in the beginning of the pandemic [69]. The European countries Spain, Germany, and France show both a similar date of the outbreak and simultaneous developments. All other states, except South Korea, show an accelerating behavior. Similar to China, South Korea managed to contain the virus by undertaking strong countermeasures.

## 5. Application to COVID-19

This section applies the methodology outlined in Section 3 to COVID-19 data. Therefore, we identify the lead-lag relations (Section 5.1) and predict future developments (Section 5.2).

### 5.1. Lead-lag Relation between Countries

Table 1 shows the pair-wise confidence intervals of the estimated lags between the top 10 countries. Overall, we observe that China’s time series is the worldwide source of the COVID-19 because all pairwise combinations with China as first country possess negative values. Therefore, China leads Italy by 31 days—in other words, Italy’s confirmed cases are around one month behind those of China. South Korea ranks second in the progression of the trend. Its curve is with China the only one that is already flattening out. All other nations are still in their growth phase. It is worth mentioning that regardless of the high number of confirmed cases, the United States is still 44 days in arrears to China. Iran is ahead of most European countries with the exception of Italy. From a European point-of-view, Italy is leading the pandemic wave. The United Kingdom and Switzerland are 12 and 44 days behind Italy and China, respectively. Hence, those countries are more likely to experience strong exponential growth in the days to come. France, Germany and Spain are despite time shifted containment actions at the same stage of the outbreak.

### 5.2. Forecast of Future Incidence in Countries

**Figure 4:**
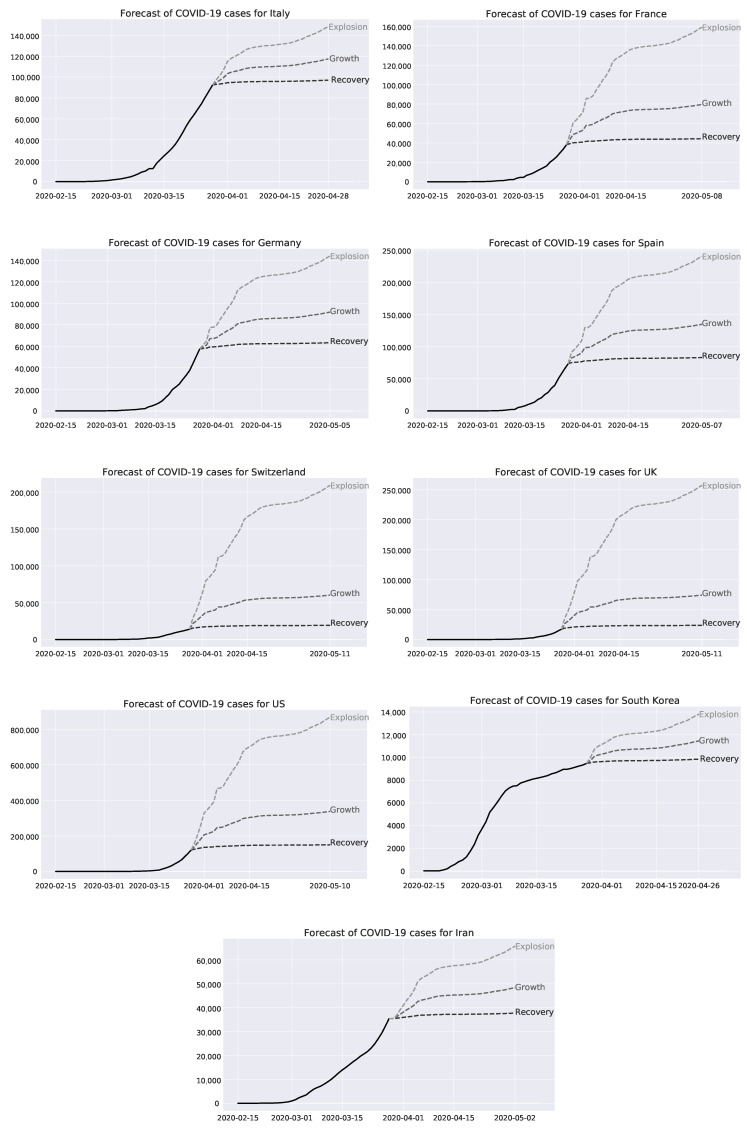
Forecast of COVID-19 cases for Italy, France, Germany, Spain, Switzerland, United Kingdom (UK), United States (US), South Korea, and Iran.

Figure 4 displays the projected development of COVID-19 cases in the top 9 countries based on China—the lead. As mentioned in Section 3, we analyze three potential scenarios, namely explosion, growth and recovery, and how they affect individual countries. At first glance, we are able to classify those countries in three stages—early (Switzerland, United Kingdom, and United States), mid (France, Germany, Spain) and late (Italy, South Korea and Iran). Countries in the first two stages are expected to face severe exponential growth in the future while late stage states can expect the curve to flatten.

Italy already faced their biggest growth. Even in the most adverse scenario, Italy’s cases would grow around 45%. This is modest compared to the other European countries and illustrates a characteristic of late stage countries. Italy is one of the most affected states. Hospitals are overwhelmed and military vehicles are in use to transport coffins to remote cremation sites as morgues collapsed under the number of Coronavirus deaths [70]. The government took several measures to contain the virus. Since 9 March 2020 Italy is in total lockdown, extending the existing Northern area restrictions [71].

The variety of outcomes of our scenarios is still wide as France is at mid stage. In the worst-case, France could see around 160,000 confirmed COVID-19 cases. Based on the growth case, this number would be slightly less than half of the explosion scenario. To slow the spread, France extended their nationwide lockdown until 15 April 2020, disrupting the daily life of 67 million people [68,72].

Germany’s healthcare system is currently preparing for a surge in COVID-19 cases. Industrial giants like Volkswagen and Daimler support the authorities with deliveries of critical medical goods, such as face masks, and the production of healthcare wares [73,74]. Despite the general preparedness, the government tries to curb the number of new infections. Hence, Germany imposed strict contact restrictions—a general lockdown is only in place in Bavaria [75,76]. Germany could still see an increase of 130% in COVID-19 cases based on the explosion case. The other scenarios predict an increase of 50% (growth) and 10% (recovery).

Lockdowns seem to be the preferred action of governments in the fight against SARS-CoV-2. Spain tightened their lockdown by closing all non-essential workplaces for two weeks as it faces a potential surge of 200% in the explosion scenario [77]. Even under the more favourable growth assumptions, cases would double. Only under recovery, the number would flatten out below 100,000. This seems fairly unrealistic as Spain keeps on topping one sad record after another. So did Spain just record a new daily death toll of 769 (date: 28 March 2020) [78].

Switzerland, the neighbour country of Italy, Germany, and France, is still at an early stage. The Swiss government declared the state of emergency on 16 March 2020 and utilized the army to support medical facilities [79,80]. Nonetheless, the low case number imply rapid growth potential. The total numbers range from 20,000 (recovery) to 200,000 (explosion), illustrating the huge variety across scenarios in an early stage nation.

In the United Kingdom, not even the Prime Minister nor the Royals are spared from COVID-19 [81,82]. The government recently joined other European governments and imposed a lockdown after less stricter actions did not play out [83]. The nation is still in the beginning of the outbreak, leaving enormous increase potential for the virus. In the growth case, the Kingdom would face around 75,000 cases while this would increase to approximately 250,000 in the explosion scenario. Such horrendous numbers would presumably lead to the breakdown of NHS, the UK’s public healthcare system.

Looking over the transatlantic, the United States faces a similar challenge as the United Kingdom. The United States is currently in a bad position to tackle the outbreak. The country is missing coordinated actions on a federal level, with each every state is undertaking its own measures. New York completely shut down while Texas’ Lieutenant Governor Dan Patrick said he is willing to die to save the economy for his grandchildren, indicating no intentions to contain SARS-CoV-2 [84,85]. The United States could see a seven fold increase in the number of cases. In the best scenario the United States confirmed COVID-19 infections could stay below 200,000. In explosion, the cases could surpass 800,000.

South Korea is with China the only country that managed to slow the spread substantially. Both countries followed well coordinated strategies but with totally different measures. China heavily restricted domestic movements and immigration while South Korea followed a softer approach. South Korea’s strategy was since the beginning of the outbreak to test as many people as quick as possible. To contain the spread at an early stage they came up with innovative ideas such as drive-through COVID-19 testing facilities and personalized text messages about nearby COVID-19 cases [86]. Through those actions, the spread has never accelerated like in the other nations. Furthermore, the government imposes mandatory quarantine and tests on all arrivals to prevent a second wave [87]. Hence, South Korea can claim the lowest number of COVID-19 cases in our sample. In all scenarios, cases stay below 15,000—a number Italy already surpassed in early March. South Korea illustrates how significant the impact of excellent disease prevention and risk management can be.

Interestingly, Iran is the only middle eastern country under the top affected countries. This seems odd as Iran is heavily sanctioned by the West which limits international interactions. The downing of Ukraine International Airlines Flight 752 in January did certainly not help either to boost international travel [88]. According to the Iranian health ministry, every 10 minutes one person dies from COVID-19 and 50 people are infected every hour [89]. The early and rapid spread of the disease in Iran is mainly caused by the close trading partnership with China, inadequate cautionary measures and a lack of drastic actions to contain the spread [90]. In our analysis, Iran could face a peak level of 65,000 (50,000) in the worst-case (mid-case) scenario.

Table 2 conducts a stress test for the countries Italy, France, Germany, Spain, Switzerland, United Kingdom (UK), United States (US), South Korea, and Iran (We thank [91] and [92] for providing the data). For this purpose, the number of intensive care unit (ICU) beds presents the upper limit of people who can be medicated in hospital. Furthermore, we calculate the number of newly incoming COVID-19 cases between 12 April 2020 and 26 April 2020 in the case of explosion. We choose this time period because this is the predicted peak of our crisis (see Figure 4). Furthermore, we assume that infected people are sick for 2 weeks [93]. According to the World Health Organization’s Regional Office for Europe, 9.90% and 47.82% of COVID-19 cases need intensive care units and hospitalization, respectively [94]. Based on those data, we create the variable “Collapse” which compares the number of ICU beds and our COVID-19 forecasts in the explosion case. Particularly, we display “Yes”, “No”, and “Unclear” by applying the following rules:

- “Yes” if there are less ICU beds than 9.90% of predicted COVID-19 cases.
- “No” if there are more ICU beds than 47.82% of predicted COVID-19 cases.
- “Unclear” if number of ICU beds falls in our margin of safety of 9.90% to 47.82% of COVID-19 cases.

First of all, we observe that the United States possesses by far the most beds (205,000)—Germany follows in second place with 24,000 beds. All other countries are equipped with less than 10,000 supply points. Six out of nine countries, namely, Italy, France, Germany, United States, South Korea and Iran, are in a position to safely survive the case explosion. Spain might be able to manage this scenario without help. United Kingdom and Switzerland would run into problems as severe COVID-19 cases, that require intensive care, exceed the number of ICU beds. Of course, a key challenge for countries with a large surface area, such as the United States, is the distribution of ICU beds. Local clusters could lead to insufficient intensive care in that area without chance to transfer patients to other hospitals that operate below capacity. This would also result in a collapse of the healthcare system.

It should be noted that the explosion scenario covers the worst case. Nevertheless, all countries should be aware that the COVID-19 pandemic confronts them with severe challenges.

## 6. Conclusions

This manuscript predicts the future spread of the Coronavirus by exploiting the identified lead-lag structure between different countries. In this respect, we make three main contributions to the existing literature. The first contribution bears on the developed statistical approach, which captures time-varying lead-lag structures between two time series. The use of dynamic time warping allows to identify similar, but time-shifted, time series. Therefore, we are able to forecast the following time sequence based on the past development of the leading time series. The second contribution relies on the application of Coronavirus infections from 1 January 2020 to 28 March 2020. We find causal relationships, e.g., China leads all other countries in the range of 29 days for South Korea and 44 days for the United States. The third contribution refers to the forecast of future COVID-19 developments based on the gained information from China. We distinguish between the scenarios recovery, growth, and explosion to guarantee the best possible disease prevention and risk management. The healthcare systems of the United Kingdom and Switzerland would collapse in case of our explosion scenario. For further investigations in this research area, hidden Markov models may be explored in order to receive probability distributions. Next, a multivariate framework could be implemented in order to account for common interactions between countries. Finally, the framework might be applied to other research areas, such as the recognition of human actions or robot programming.

## Figures and Tables

**Table 1:** Pair-wise confidence intervals of the estimated lags between the countries. Negative values indicate that the first country (row) leads the second country (column) and vice versa.

	China	Italy	France	Germany	Spain	Switzerland	UK	US	S. Korea	Iran
China	-	(−44, −18)	(−64, −18)	(−50, −26)	(−57, −23)	(−60, −28)	(−59, −29)	(−59, −29)	(−37, −21)	(−46, −24)
Italy	-	-	(−11, −7)	(−8, −8)	(−10, −8)	(−14, −10)	(−15, −9 )	(−13, −11)	(−3, 7)	(−7, −1)
France	-	-	-	(0, 0)	(0, 0)	(−4, −2)	(−3, −3)	(−3, −1)	(7, 15)	(3, 7)
Germany	-	-	-	-	(−2, 0)	(−5, −3)	(−5, −3)	(−3, −3)	(9, 15)	(2, 6)
Spain	-	-	-	-	-	(−4, −2)	(−4, −2)	(−3, −1)	(9, 13)	(5, 9)
Switzerland	-	-	-	-	-	-	(0, 0)	(−1, 3)	(16, 24)	(12, 16)
UK	-	-	-	-	-	-	-	(1, 1)	(14, 26)	(11, 17)
US	-	-	-	-	-	-	-	-	(12, 16)	(6, 10)
S. Korea	-	-	-	-	-	-	-	-	-	(−8, −4)
Iran	-	-	-	-	-	-	-	-	-	-

**Table 2:** Population, intensive care unit (ICU) beds, forecast of newly incoming COVID-19 cases between 12 April 2020 and 26 April 2020, and if there is a collapse in the healthcare system of Italy, France, Germany, Spain, Switzerland, United Kingdom (UK), United States (US), South Korea, and Iran.

Country	Population	ICU Beds	COVID-19 Cases Explosion	Collapse
Italy	60,431,280	7500	14,258	No
France	66,987,240	7500	14,804	No
Germany	82,927,920	24,000	9540	No
Spain	46,723,750	5000	20,039	Unclear
Switzerland	8,516,540	1000	40,346	Yes
UK	66,488,990	4500	49,621	Yes
US	327,167,430	205,000	138,211	No
South Korea	51,635,260	5500	1620	No
Iran	81,800,270	4000	4442	No
